# White matter integrity mediates the associations between white matter hyperintensities and cognitive function in patients with silent cerebrovascular diseases

**DOI:** 10.1111/cns.14015

**Published:** 2022-11-22

**Authors:** Jing Chen, Anyan Ge, Ying Zhou, Yuanyuan Ma, Shaoping Zhong, Caizhong Chen, Weibin Shi, Jing Ding, Xin Wang

**Affiliations:** ^1^ Department of Neurology, Zhongshan Hospital Fudan University Shanghai China; ^2^ Department of Neurology, XiaMen Branch, Zhongshan Hospital Fudan University Shanghai China; ^3^ Department of Radiology, Zhongshan Hospital Fudan University Shanghai China; ^4^ Health Examination Center, Zhongshan Hospital Fudan University Shanghai China; ^5^ Department of the State Key Laboratory of Medical Neurobiology and MOE Frontiers Center for Brain Science，Institutes of Brain Science Fudan University Shanghai China

**Keywords:** diffusion tensor imaging, execution function, information processing speed, mediation analysis, white matter hyperintensities

## Abstract

**Objective:**

To evaluate the relationships between cognitive function and white matter hyperintensity volume (WMHV) in patients with silent cerebrovascular disease and to investigate whether white matter integrity or brain atrophy play a role in this association.

**Methods:**

Automated Fiber Quantification and Voxel‐ based morphometry were used to track and identify the integrity of 20 well‐defined white matter tracts and to measure the gray matter volume (GMV). A linear regression model was applied for examining the associations between cognitive function and WMHV and mediation analysis was used to identify the roles of white matter integrity or GMV in the influence of WMHV on cognitive function.

**Results:**

Two hundred and thirty‐six individuals were included for analysis. Executive function was linearly associated with fractional anisotropy (FA) of the right interior frontal occipital fasciculus (IFOF) (*β* = 0.193; 95% CI, 0.126 to 1.218) and with WMHV (*β* = −0.188; 95% CI, −0.372 to −0.037). Information processing speed was linearly associated with WMHV (*β* = −0.357; 95% CI, −0.643 to −0.245), FA of the right anterior thalamic radiation (ATR) (*β* = 0.207; 95% CI, 0.116 to 0.920), and FA of the left superior longitudinal fasciculus (SLF) (*β* = 0.177; 95% CI, 0.103 to 1.315). The relationship between WMHV and executive function was mediated by FA of the right IFOF (effect size = −0.045, 95% CI, −0.015 to −0.092). Parallel mediation analysis showed that the association between WMHV and information processing speed was mediated by FA of the right ATR (effect size = −0.099, 95% CI, −0.198 to −0.038) and FA of the left SLF (effect size = −0.038, 95% CI, −0.080 to −0.003).

**Conclusion:**

These findings suggest a mechanism by which WMH affects executive function and information processing speed by impairing white matter integrity. This may be helpful in providing a theoretical basis for rehabilitation strategies of cognitive function in patients with silent cerebrovascular diseases.

## Graphical abstract

These findings may provide an underlying mechanism that white matter hyperintensities affects executive function and information processing speed via impairing the white matter integrity. It may be helpful in providing theoretical basis for rehabilitation strategies of cognitive function in patients with silent cerebrovascular diseases.

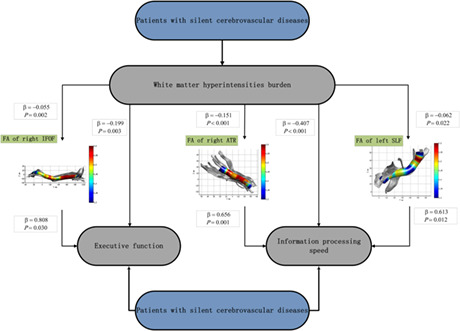

## 1 INTRODUCTION

Silent cerebrovascular diseases identified by neuroimaging are very common in older people.^1^ White matter lesions, also called white matter hyperintensities (WMHs), are evidenced by speckled or patchy white matter changes in the subcortical or periventricular areas.^2^ The histopathologic changes in WMHs are nonspecific and characterized by demyelination, Wallerian degeneration, neuron loss, oligodendroglial apoptosis, and gliosis.^3^, ^4^ WMHs have been regarded as an important symptom of silent cerebrovascular diseases and are correlated with other cardiovascular risk factors such as advanced age, hypertension, diabetes mellitus, and smoking.^5^ Furthermore, accumulating evidence indicates that WMHs are related to impairment in multiple cognitive domains.^6^, ^7^, ^8^, ^9^ However, the results of these studies are somewhat contradictory and require careful consideration. First, these studies examining WMH‐related cognitive impairments have had small participant samples and thus might be insufficiently powered to detect cognitive deficits. Second, a wide range of cognitive domains including executive function, information processing speed, episodic memory, working memory, and visual–spatial ability are needed to investigate the relationships between WMHs and cognitive function. Third, the underlying mechanisms mediating the association between WMHs and cognitive function in these patients need to be further investigated.

Diffusion tensor imaging (DTI) is a new noninvasive magnetic resonance imaging (MRI) technique that can track the fiber tracts and can serve as an effective method for the quantification of the diffusion of water in tissues in living human brains.^10^ Previous studies based on DTI data have shown that the integrity of the white matter is damaged and that the extent of damage is related to cognitive impairment in patients with WMHs.^11^, ^12^ Because WMHs often coexist with brain atrophy in older adults, understanding the correlation between WMHs and brain structure might be helpful in clarifying the mechanisms through which WMHs affect cognitive impairment. Voxel‐ based morphometry (VBM) is an automated technique widely used for quantitative measurements of brain volume.^13^ Studies have shown that patients with WMHs have significantly decreased gray matter volume (GMV) in some cognition‐related brain regions such as the left middle frontal gyrus, the right middle temporal gyrus, and the right angular gyrus. Further, it has been reported that a greater total WMH volume (WMHV) is associated with smaller GMV and that smaller GMV is correlated with lower levels of cognitive domains such as episodic memory.^14^, ^15^ These findings suggest that the mechanisms through which WHMs results in cognitive disorders might be associated with attenuated conduction in the fasciculus due to the impairment of white matter integrity or brain atrophy due to decreased GMV.

In the current study, we first examined the relationships between cognitive function as measured by a range of cognitive domains, total WMHV, the integrity of white matter tracts, and total GMV in patients with silent cerebrovascular diseases. We then investigated whether the extent of white matter integrity or brain atrophy played roles in the influence of WMHV on cognitive function. We used the Automated Fiber Quantification (AFQ) approach to automatically track and identify 20 well‐defined white matter tracts defined by waypoint regions of interest. This tract‐oriented method can correct the delineation of white matter tracts, provide profiles of diffusion parameters for each tract, and maintain anatomical correspondence.^16^ We applied diffusion parameters and fractional anisotropy (FA) to study microstructural white matter integrity as reported previously.^17^ In light of the previous findings described above, we hypothesized that reduced cognitive function, especially in executive function and information processing speed, is associated with larger total WMHV. We used parallel mediation models to test the hypothesis that white matter integrity or GMV mediates the potential associations between WMHV and cognitive function.

## 2 MATERIALS AND METHODS

### 2.1 Participants

This study was reviewed and approved by the institutional review board of Zhongshan Hospital Affiliated to Fudan University, and informed consent was obtained from all participants. A consecutive series of subjects without overt cognitive impairment who underwent physical examinations in Zhongshan Hospital Affiliated to Fudan University from March 2018 to February 2020 were screened for inclusion by a consensus panel of three senior neurologists and two radiologists. The inclusion criteria were as follows: (1) aged 50–85 years; (2) no overt clinical signs or positive symptoms of clinical manifestations upon neurophysical examination; (3) any degree of WMH changes in the subcortical or periventricular areas as seen on T2‐weighted images (T2WI) or fluid attenuated inversion recovery (FLAIR) images from MRI; (4) being able to cooperate with MRI examination and neuropsychological scale tests; and (5) written informed consent. The exclusion criteria included (1) aged <50 or >85 years; (2) other brain abnormalities or psychiatric diseases or clinically significant or unstable medical diseases; (3) use of medications that might affect cognitive function, such as antipsychotics and antiepileptics; (4) leukoencephalopathy of nonvascular origin (immunological demyelinating, metabolic, toxic, infectious, other); and (5) contraindications for MRI scanning or claustrophobia.

The baseline clinical assessments included demographic characteristics (e.g., age and gender) and the patient's education history. The standard vascular risk factors included body mass index, hypertension, diabetes mellitus, dyslipidemia, cigarette smoking, and alcohol use.

### 2.2 Neuropsychological measurement

The Mini‐Mental State Examination (MMSE) and the Montreal Cognitive Assessment (MoCA) were employed as global cognitive screening methods. An extensive neuropsychological test battery was administered by trained neuropsychologists covering the following cognitive domains: (1) executive function ‐ trail‐making test part B, inverted digit span test, and Stroop C; (2) information processing speed ‐ trail‐making test part A and Stroop B; (3) episodic memory ‐ auditory verbal learning test part N4 and N5; (4) working memory ‐ sequential digit span test; and (5) visual–spatial ability ‐ clock drawing test. For every neuropsychological test, we used z‐scores to calculate compound measures. The z‐scores of cognitive tests in which a higher score indicated worse performance were inverted (−z) for the calculation of compound measure scores. For each individual participant, neuropsychological measurements were scheduled to be completed on the same day as the brain MRI scan and within 1 week after enrollment.

### 2.3 MRI scanning

The MRI data were acquired via a 3.0T GE Discovery MR750 scanner with a 32‐channel head coil. During scanning, all participants were instructed to remain awake, keep their eyes closed, remain motionless, and attempt to think of nothing. Tight but comfortable foam padding was used to minimize head motion, and earplugs were used to reduce scanner noise. The imaging protocols included the following parameters: (1) T2WI were obtained by a single shot fast spin echo (FSE) sequence: repetition time (TR) = 5500 ms, repetition echo time (TE) = 90 ms, field of view (FOV) = 256 mm × 256 mm, matrix = 256 × 256, slice thickness = 4 mm, gap = 1 mm; (2) T2 FLAIR images were obtained with a single shot fast spin echo sequence: TR = 5500 ms, TE = 97 ms, FOV = 256 mm × 256 mm, matrix = 256 × 256, slice thickness = 5 mm, number of slices =22, and gap = 1 mm; (3) high‐resolution sagittal T1WI were acquired by a three‐dimensional magnetization‐prepared rapid gradient echo sequence: TR = 7.4 ms, TE = 3.1 ms, FA = 11°, FOV = 256 mm × 256 mm, matrix = 256 × 256, slice thickness = 1 mm, voxel size = 1 mm × 1 mm × 1 mm, NEX = 1, and number of slices = 196; and (4) DTI images were obtained with a diffusion‐weighted pulsed‐gradient spin echo‐planar imaging sequence: TR = 8500 ms, TE = 94.6 ms, FA = 90°, FOV = 256 mm × 256 mm, matrix = 128 × 128, slice thickness = 2 mm, number of slices = 64, and 61 different diffusion directions for the diffusion‐sensitizing gradients at a *b*–value of 2000 s/mm^2^.

### 2.4 DTI processing and automatic tract identification analysis

**FIGURE 1:**
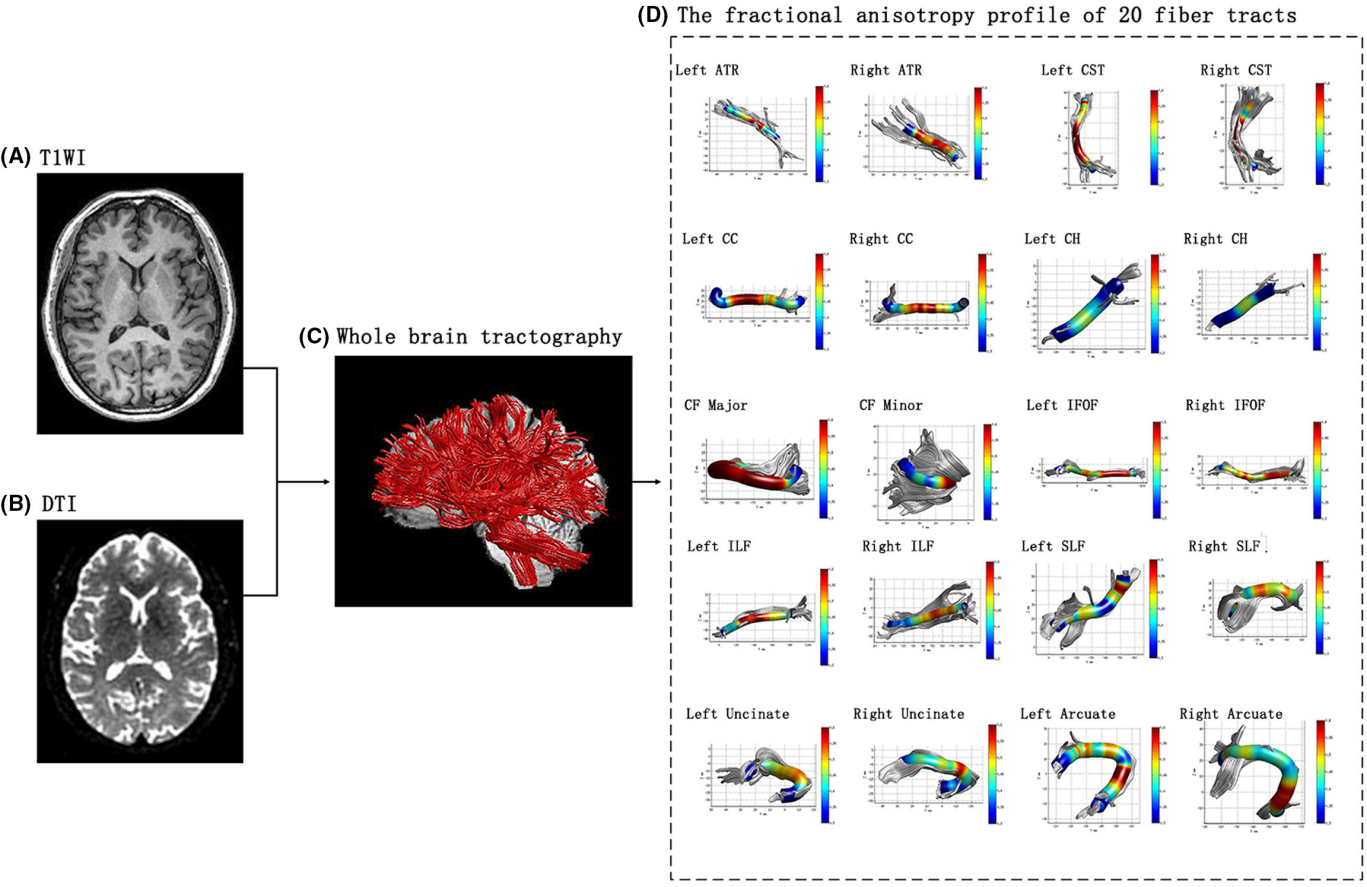
The flowchart of the calculation of diffusion measures and the track profiles of FA value in 20 fiber tracts. (A) T1WI; (B) DTI; (C) the result of the whole brain tractography; (D) the fraction anisotropy profile of 20 fiber tracts. ATR, Anterior Thalamic Radiation; CC, Cingulum Cingulate; CFJ, Callosum Forceps Minor; CFM, Callosum Forceps Major; CH, Cingulum Hippocampus; CST, Corticospinal tract; DTI, diffusion tensor imaging; FA, fraction anisotropy; IFOF, Inferior Fronto‐ occipito Fasciculus; ILF, Inferior Longitudinal Fascicle; SLF, Superior Longitudinal Fascicle; T1WI, T1‐weighted images.

Diffusion tensor imaging data preprocessing was implemented with FSL v5.0 software (Oxford Center for Functional MRI of the Brain, http://www.fmrib.ox.ac.uk/fsl/), and data were manually checked for excessive dropped volumes and imaging artifacts. The FMRIB's Diffusion Toolbox v3.0 was used to correct for eddy currents and head motions and for calculating the diffusion measures (FA). The AFQ package (http://www.jasonyeatman.com/software/) was used to identify the 20 main whole‐brain fibers, including the left and right Anterior Thalamic Radiation (ATR), left and right Corticospinal tract (CST), left and right Cingulum Cingulate (CC), left and right Cingulum Hippocampus (CH), Callosum Forceps Major (CFM), Callosum Forceps Minor (CFJ), left and right Inferior Fronto‐occipito Fasciculus (IFOF), left and right Inferior Longitudinal Fascicle (ILF), left and right Superior Longitudinal Fascicle (SLF), left and right Uncinate Fascicle (UF), and left and right Arcuate Fascicle (AF). The diffusion measures along the tract trajectory were quantified in each participant's brain. Each tract was sampled into 100 equidistant nodes, and the ‘tract profile’ of each fiber tract was created by mapping the diffusion measures onto each tract along the central portion of the tract. In the present study, the middle 80% of the fibers were selected to avoid the influence from crossing fibers near cortical terminations and from the potential partial volume effect at the gray matter or white matter border.^17^ The mean tract values of the FA for each participant for each fiber were then used for analysis. Figure 1 shows the flowchart for calculating the diffusion measures and the track profiles of the FA value in 20 fiber tracts for one participant. In the current study, DTI data from eight participants failed to successfully align. Detailed information about the successful identification rate for each of the 20 fiber tracts in the participants is given in Appendix 1: Table A1.

### 2.5 Voxel‐based morphometry (VBM) analysis

The individual three‐dimensional T1WI preprocessing was conducted using the Statistical Parametric Mapping (SPM) 12 software packages (https://www.fil.ion.ucl.ac.uk/spm/software/spm12/), and the optimized VBM analysis was performed using the CAT12 toolbox (http://www.neuro.uni‐jena.de/cat/). The VBM analysis procedure in the present study included a quality check; normalization by the Diffeomorphic Anatomical Registration Through Exponentiated Lie Algebra method using the IXI550_MNI 152 template; segmentation into gray matter, white matter, and cerebrospinal fluid; modulation by non‐linear components to compensate for the loss of information in absolute volume through spatial normalization; data quality checks, and smoothing with a Gaussian kernel of 8 mm full width at half‐maximum. Statistical analyses were performed on the smoothed images generated during the final preprocessing step.

### 2.6 Analysis of WMHV and GMV

WMH was defined as hyperintense lesions without central hypointensity in the cerebral white matter on the FLAIR images, which differentiates these WMHs from lacunar infarcts or perivascular spaces.^18^ WMHs located in the cerebellum or brain stem were excluded. The semi‐automated freeware NeuRoi (http://www.nottingham.ac.uk/research/groups/clinicalneurology/neuroi.aspx) was used for volumetric analysis of the WMH and to calculate the total brain volume (TBV). All WMHs for each participant were outlined slice by slice independently by two neurologists (both with more than 8 years of experience) who were blinded to other clinical data. We used the average total WMHV calculated by these two researchers for further analyses. The procedure was as follows. First, WMHs were semi‐automatically drawn by manually adjusting the signal‐intensity ranges. The lesions were then interconnected between the adjacent axial FLAIR images and were combined into one object. The total WMHV was automatically calculated by adding the volumes of each participant.^19^ Finally, the total WMHV was normalized to the TBV. The WMHV was first divided by the TBV and then log‐transformed to better approximate a normal distribution before inclusion in the analyses.^20^

The GMVs were calculated from the individual smoothed images for each participant. The overall GMV (including the cortical gray matter, thalamus, caudate nucleus, putamen, pallidum, amygdala, and accumbens area) was also acquired using the NeuRoi software. Similarly, the GMV was normalized to the TBV.

### 2.7 Lesion overlap map

White matter hyperintensities for each participant were outlined slice by slice on T2 FLAIR images and a lesion mask for each patient was generated. After spatial normalization to the Montreal Neurological Institute space, all of the patients' lesion masks overlapped. Then the individual lesion masks were averaged and overlaid using a MNI152_T2 template to create a lesion overlap map.

### 2.8 Statistical analysis

The normality of the data distribution was evaluated by the Kolmogorov–Smirnov method and histogram inspection. Data are reported as the mean and standard deviation or as the median and interquartile range for continuous variables and as the number and percentage for categorical variables. Skewed variables (WMHV and GMV) were log10 transformed, and missing data were dealt with using the mean value interpolation method. All analyses were conducted using Statistical Package for the Social Sciences (SPSS 24.0), and *p* < 0.05 was considered to indicate a significant difference.

Associations between the cognitive function variables (executive function, information processing speed, episodic memory, working memory, and visual–spatial ability) and the log transformed WMHV were examined using multivariate linear regression models by stepwise methods. Variables included were sociodemographic variables (sex, age, educational level, smoking, and drinking), clinical indices (BMI, hypertension, diabetes mellitus, and dyslipidemia), and brain structural neuroimaging indicators (the mean FA of 20 white matter tracts and the GMV). The variance inflation factor was applied to determine the multicolinearity between variables. The variables (log‐transformed WMHV and the neuroimaging indicators) that were included in a linear regression model were further used for mediation analysis.

In the current study, we applied a newly developed mediation analysis method using PROCESS (http://www.processmacro.org/index.html) installed in SPSS 24.0.^21^ This up‐to‐date method has the advantage that there is no dependence on the mediating effect of X on Y when testing the significance of each path (a, b, and c). Alternatively, only the significance of mediation path a * b needs to be tested.^22^ In mediation analysis, cognitive function was the dependent variable, WMHV (normalized and log transformed) was the independent variable, and the mean FA of 20 white matter tracts or GMV was the mediator variable. All analyses were corrected for sex, age, education level, smoking, drinking, BMI, hypertension, diabetes mellitus, and dyslipidemia. The standard error parameters of the mediation models were bootstrapped. Parameters of the mediation models were as follows: bootstrap sample = 5000, model number = 4, bootstrap confidence interval (CI) method = bias corrected, confidence level for CI = 95%. The effect size, bootstrap standard error, bootstrap lower limit of confidence interval, bootstrap upper limit of confidence interval, and the proportion of relative effects of the total effect, direct effect, and mediating effect were reported.

## 3 RESULTS

### 3.1 Demographic and clinical characteristics

**FIGURE 2:**
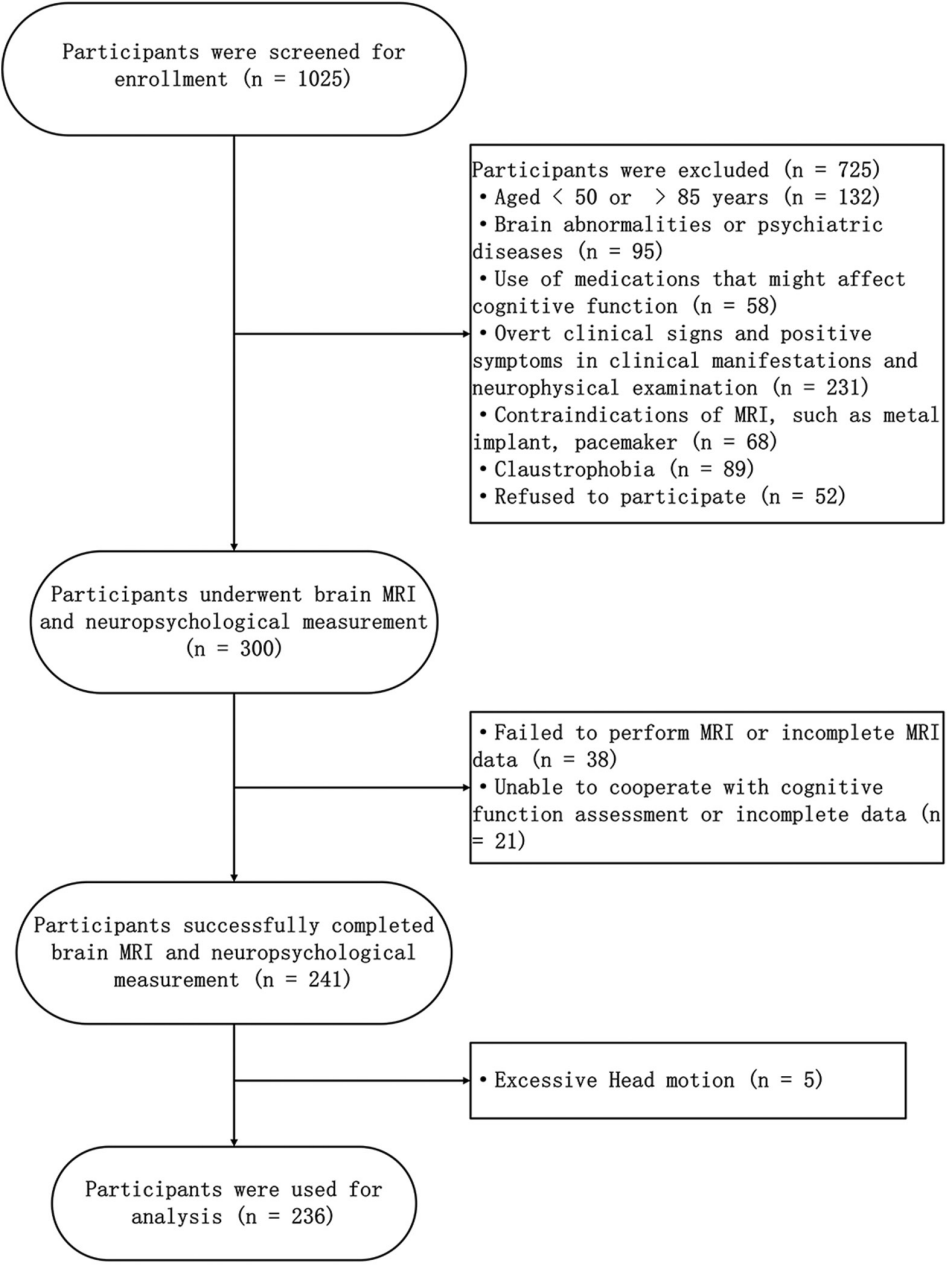
Flowchart of the study population.

**FIGURE 3:**
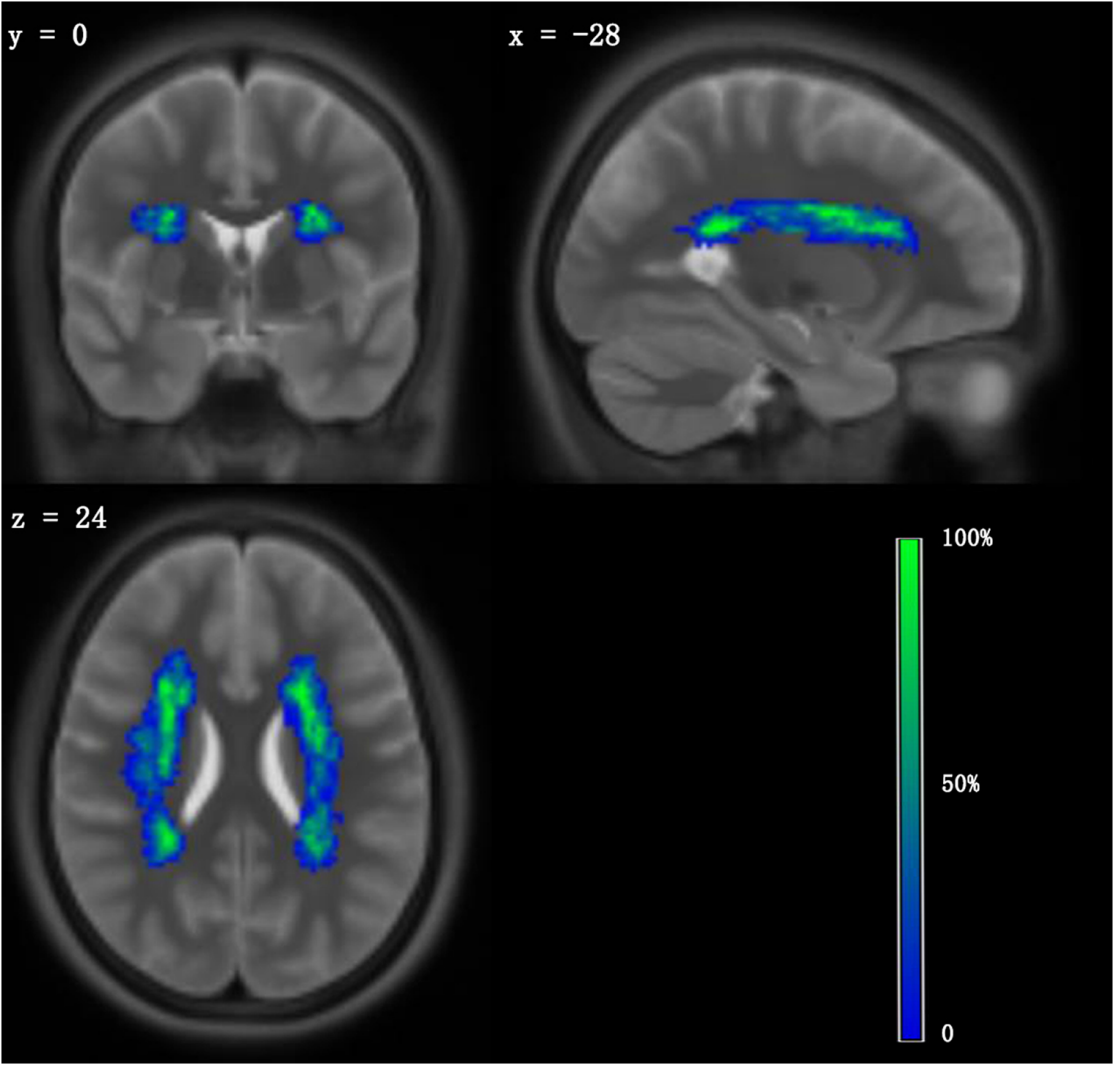
Lesion overlap map across participants. Lesion maps were normalized to an MNI reference brain. The color bar indicates the percentage of lesion overlap.

We screened 1025 potential participants for enrollment. Among them, 673 were excluded because they did not satisfy the inclusion and exclusion criteria, and 52 subjects refused to participate for various reasons. There were only 241 subjects who successfully completed head MRI scanning and neuropsychological measurement. Five participants were excluded because of excessive head motion during the scanning. Finally, 236 individuals were used for analysis. The flowchart of the study population is shown in Figure 2. The overall mean age was 56.14 ± 8.2 years, and 145 (61.4%) of them were male. The mean level of education was 9 years with a range of 7 to 12 years. The participants had an average BMI of 24.59 kg/m^2^, MMSE of 28.17, and MoCA of 23.83. Hypertension, diabetes mellitus, and dyslipidemia were seen in 99 (41.9%), 35 (14.8%), and 126 (53.4%) of the participants, respectively. A total of 107 (45.3%) and 112 (47.5%) individuals had smoked and drank alcohol, respectively. The median compound measure scores of executive function, information processing speed, episodic memory, working memory, and visual–spatial ability were 0.08, 0.13, −0.04, 0.17, and 0.29, respectively. The median percentage normalized WMHV was 4.22 × 10^−4^ with a range of 2.10 × 10^−4^ to 8.69 × 10^−4^, and the median percentage normalized GMV was 3.71 × 10^−1^ with a range of 2.50 × 10^−1^ to 4.04 × 10^−1^ (Table 1). The reproducibility for measuring WMHV, as determined by recalculating 50 randomly allocated FLAIR images, was 0.998 (95% CI, 0.996–1.000). The WMH lesion overlap map of the study sample is shown in Figure 3.

**TABLE 1:** Demographics, clinical, cognitive and radiologic profiles of the study population.

	Total
No.	236
Age^a^, years	56.14 ± 7.85
Sex^b^, male	145 (61.4)
Educational level^c^, years	9.00 [7.00–12.00]
BMI^a^, kg/m^2^	24.59 ± 3.28
Smoking^b^	107 (45.3)
Drinking^b^	112 (47.5)
Hypertension^b^	99 (41.9)
Diabetes mellitus^b^	35 (14.8)
Dyslipidemia^b^	126 (53.4)
MMSE^a^	28.17 ± 1.49
MoCA^a^	23.83 ± 3.54
Executive function^c^	0.08 [−0.44–0.51]
Information processing speed^c^	0.13 [−0.48–0.41]
Episodic memory^c^	−0.04 [−0.78–0.69]
Working memory^c^	0.17 [−0.53–0.88]
Visual–spatial ability^c^	0.29 [−0.41–0.32]
Percentage normalized WMH volume (×10^−4^)^c^	4.22 [2.10–8.69]
Percentage normalized GM volume (×10^−1^)^c^	3.71 [2.50–4.04]

Abbreviations: BMI, body mass index; GM, gray matter; MMSE, Mini‐mental State Examination; MoCA, Montreal cognitive assessment; WMH, white matter hyperintensity.

^a^ Data are showed as mean ± standard deviation.

^b^ Data are showed as number of patients, with parentheses in percentages.

^c^ Data are showed as median with interquartile range in brackets.

### 3.2 Association between cognitive function and WMH burden

In linear stepwise regression analyses, executive function was positively associated with the mean FA value of the right IFOF (*β* = 0.193; 95% CI, 0.126 to 1.218; *p* = 0.016), negatively associated with log WMHV (*β* = −0.188; 95% CI, −0.372 to −0.037; *p* = 0.017), and positively associated with education level (*β* = 0.329; 95% CI, 0.026 to 0.072; *p* < 0 0.001) (Table 2). Information processing speed was linearly associated with log transformed WMHV (*β* = −0.357; 95% CI, −0.643 to −0.245; *p* < 0 0.001), the mean FA value of the right ATR (*β* = 0.207; 95% CI, 0.116 to 0.920; *p* = 0.012), and the mean FA value of left SLF (*β* = 0.177; 95% CI, 0.103 to 1.315; *p* = 0.022) (Table 2). Variance inflation factor values were less than 1.20 for every factor included in the linear regression analysis. However, we did not find any association between the other compound measure scores (episodic memory, working memory and visual–spatial ability) and log transformed WMHV. Also, there was no significant correlation between the five cognitive domains and GMV.

**TABLE 2:** Linear regression analysis for executive function and information processing speed.

Variables	Unstandardized coefficient	Standardized coefficient	*T*	Boot 95% CI		*p*	VIF
Executive function							
Constant	−1.840	–	−5.653	−2.485	−1.196	0.000	–
*p*	VIF	0.329	4.138	0.026	0.072	0.000	1.104
FA of right IFOF	0.672	0.193	2.436	0.126	1.218	0.016	1.097
Log_10_^(WMHV)^	−0.205	−0.188	−2.413	−0.372	−0.037	0.017	1.062
Information processing speed							
Constant	−2.188	–	−6.582	−2.846	−1.530	0.000	‐
Log_10_^(WMHV)^	−0.444	−0.357	−4.411	−0.643	−0.245	0.000	1.178
FA of right ATR	0.518	0.207	2.548	0.116	0.920	0.012	1.191
FA of left SLF	0.711	0.177	2.315	0.103	1.319	0.022	1.058

Abbreviations: ATR, anterior thalamic radiation; CI, confidence interval; FA, fractional anisotropy; IFOF, interior frontal occipital fasciculus; SLF, superior longitudinal fasciculus; VIF, Variance inflation factor; WMHV, white matter hyperintensity volume.

### 3.3 Mediating role of white matter integrity between cognitive function and WMH burden

**FIGURE 4:**
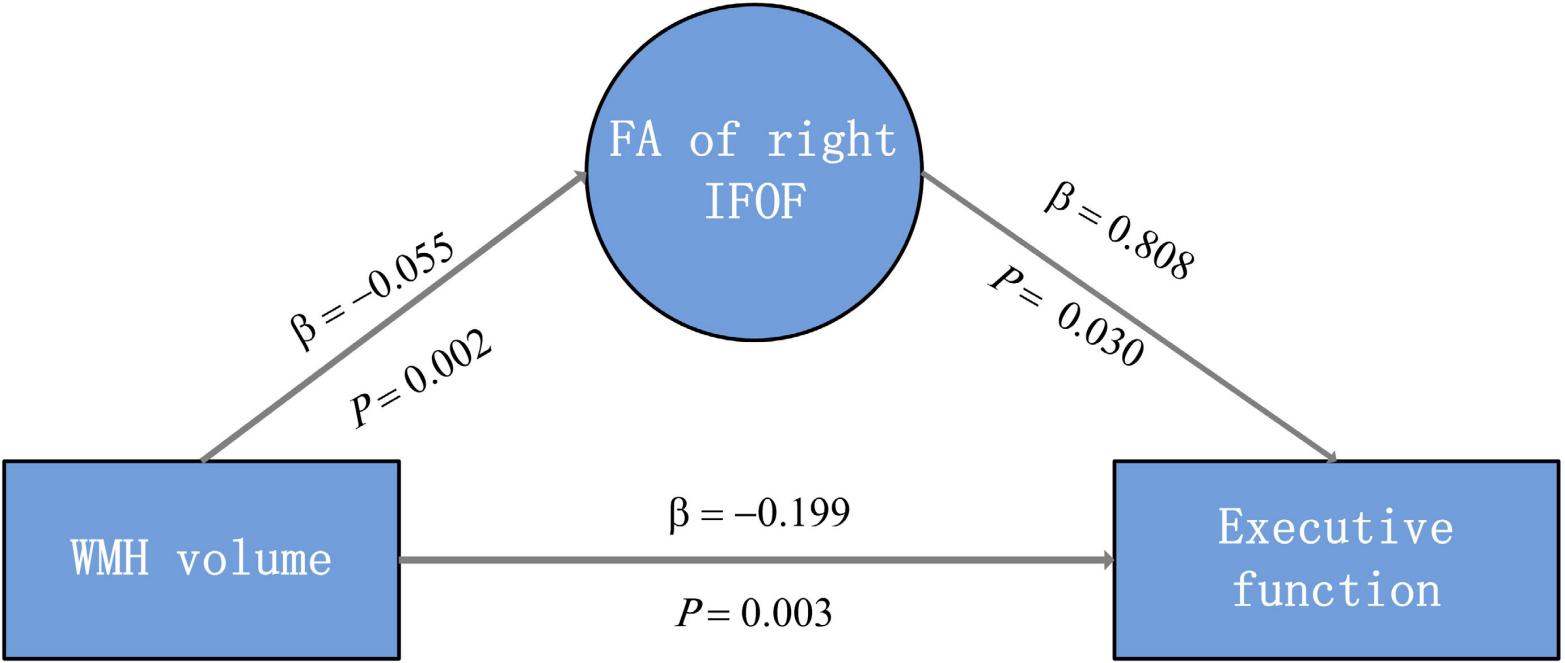
The mediating effect of the mean FA value of the right IFOF on the association between WMH volume and executive function. FA, fraction anisotropy; IFOF, Inferior Fronto‐ occipito Fasciculus.

**FIGURE 5:**
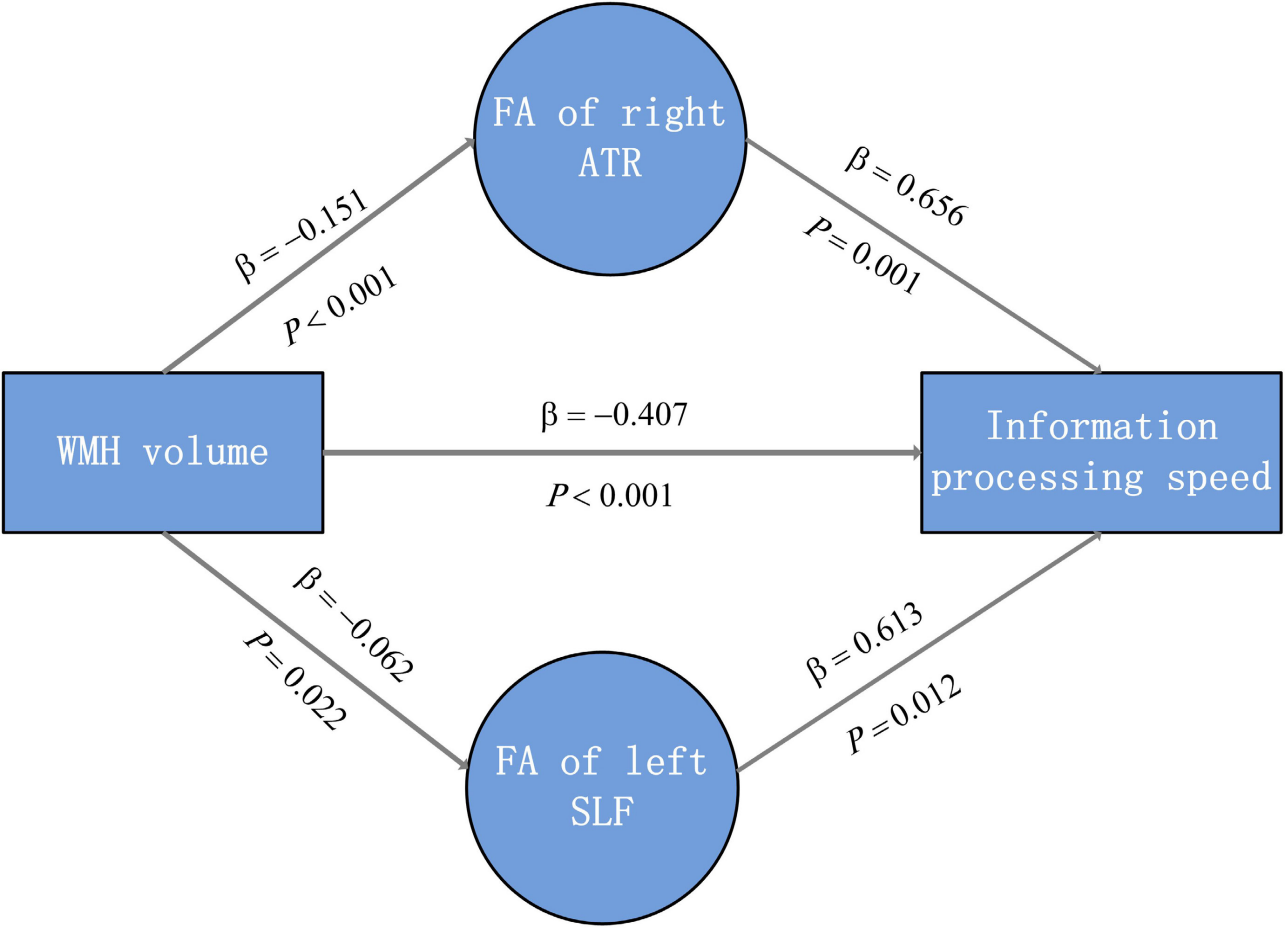
The mediating effects of the mean FA value of the right ATR and the left SLF on the association between WMH volume and information processing speed. ATR, Anterior Thalamic Radiation; FA, fraction anisotropy; SLF, Superior Longitudinal Fascicle.

Mediation analysis showed a significant indirect effect from WMHV on executive function mediated by the mean FA of the right IFOF (effect size = −0.045, 95% CI, −0.015 to −0.092, proportion of relative effect = 22.61%) (Table 3 and Figure 4). Parallel mediation analysis showed a significant indirect effect of WMHV on information processing speed mediated by the mean FA of the right ATR (effect size = −0.099, 95% CI, −0.198 to −0.038, proportion of relative effect = 24.32%) and by the mean FA of the left SLF (effect size = −0.038, 95% CI, −0.080 to −0.003, proportion of relative effect = 9.34%) (Table 4 and Figure 5). The detailed results of the mediation analysis are listed in Appendix 2: Table A2 and Appendix 3: Table A3.

**TABLE 3:** Total effect, direct effect and mediating effect of log transformed WMH volume on executive function.

	Effect size	Boot SE	Boot LLCI	Boot ULCI	Proportion of relative effect
Total effect	−0.199	0.066	−0.3315	−0.0035	
Direct effect	−0.154	0.065	−0.288	−0.023	77.39%
Mediating effect of FA of right IFOF	−0.045	0.018	−0.015	−0.092	22.61%

Abbreviations: FA, fractional anisotropy; IFOF, interior frontal occipital fasciculus; LLCI, Lower limit of confidence interval; SE, standard error; ULCI, Upper limit of confidence interval.

**TABLE 4:** Total effect, direct effect and mediating effect of log transformed WMH volume on information processing speed.

	Effect size	Boot SE	Boot LLCI	Boot ULCI	Proportion of relative effect
Total effect	−0.407	0.094	−0.591	−0.222	
Direct effect	−0.270	0.096	−0.459	−0.081	66.34%
Total indirect effect	−0.137	0.048	−0.062	−0.254	33.66%
Mediating effect of FA of right ATR	−0.099	0.041	−0.198	−0.038	24.32%
Mediating effect of FA of left SLF	−0.038	0.019	−0.080	−0.003	9.34%
Effect of FA of right ATR minus FA of left SLF	−0.061	0.040	−0.156	0.003	–

Abbreviations: FA, fractional anisotropy; LLCI, Lower limit of confidence interval; SE, standard error; SLF, superior longitudinal fasciculus; TR, anterior thalamic radiation; ULCI, Upper limit of confidence interval.

### 3.4 Verification analysis

The mean tract values of the FA of the 20 fibers were reanalyzed for all participants by selecting the middle 60% of the fibers and 100% of the fibers, respectively, to confirm the reproducibility of the data. The results were similar to the main findings in multivariate linear regression and mediation analyses. Regardless of selecting the middle 60% of the fibers or 100% of the fibers, executive function was associated with the mean FA value of the right IFOF and WMHV; information processing speed was correlated with the mean FA value of the right ATR, mean FA value of the left SLF, and the WMHV (Appendix 4: Tables A4 and A5); the mean FA of the right IFOF mediated the association between WMHV and executive function (Appendix 5: Tables A6 and A7); and the mean FA of the right ATR and the left SLF mediated the association between WMHV and information processing speed (Appendix 6: Tables A8 and A9).

## 4 DISCUSSION

In this study, we explored the relationships between cognitive domains (executive function, information processing speed, episodic memory, working memory, and visual–spatial ability), the WMH burden, white matter integrity as measured by the FA value, and brain atrophy as measured by GMV in patients with silent cerebrovascular diseases. The strengths of the current study include its comprehensive and detailed neuropsychological measurements and quantification of the WMH burden among patients with silent cerebrovascular diseases. In addition, 20 well‐defined white matter tracts in the whole brain were tracked by the AFQ method, and their integrity was quantified by FA. The up‐to‐date mediation model used in the current study is helpful in clarifying the direct effect of the WMH burden on specific cognitive domains and the indirect effect mediated by white matter integrity.

We found that executive function was positively associated with the mean FA of the right IFOF and negatively associated with the log‐transformed WMHV. Significant correlations were identified between the information processing speed and the WMHV and the mean FA of the right ATR and the left SLF. Furthermore, we observed a direct negative effect of the WMH on executive function and an indirect effect of the WMH on executive function by impairing the integrity of the right IFOF. Meanwhile, we found a positive correlation between educational level and executive function as seen in other studies.^23^, ^24^ Because the purpose of the present study was not to investigate whether education level acts as a factor in mediating WMH via executive function, education level was adjusted in the mediation analysis as a covariate.

We found that the WMHs affected information processing speed not only in a direct way, but also indirectly by impairing the integrity of the right ATR and the left SLF. Our findings imply that the WMHs in patients with silent cerebrovascular diseases play a negative role in execution function and information processing speed, and these influences were partially dependent on the integrity of the right IFOF, right ATR, and left SLF. According to this finding, rehabilitation methods targeted at repairing white matter fibers may improve cognitive function in WMH patients. Our results are thus likely to be helpful in providing a theoretical basis for rehabilitation strategies of cognitive function in patients with silent cerebrovascular diseases.

Our findings are consistent with previous studies suggesting that WMHs in patients without overt cognitive impairment may eventually lead to cognitive impairment and even dementia.^25^, ^26^ The results of the current study demonstrated that WMHs may affect cognitive function by reducing the integrity of white matter tracts and not by inducing brain atrophy. Previous studies have shown that the effect of white matter injury on cognitive function may be caused by cortical atrophy.^27^ The mechanism behind this effect is demyelination of the white matter that further leads to slight axonal injury but which can also affect the neuronal body via Wallerian degeneration.^28^ Injury and death of large numbers of neuronal bodies will result in brain atrophy.^29^ In our study, because we included patients with silent cerebrovascular diseases without overt cognitive impairment, it is possible that the white matter injury caused by WMH was not so serious as to cause axonal injury. Thus, we failed to identify any association between cognitive domain scores and GMV.

In a recent study, the authors found that normal cognition group with WMHs showed significantly decreased FA in the bilateral ATR and CFJ compared to the healthy control group. Results from that cross‐sectional study demonstrated that there were microstructural changes in the white matter in patients with WMH even without significant cognitive decline.^30^ Unfortunately, that study did not investigate the correlation between the white matter integrity and cognitive function among patients with normal cognitive performance. The results from our population of patients with silent cerebrovascular disease did not show significant correlations between WMHs and memory‐related cognitive functions, which is in line with previous studies.^31^, ^32^ These studies indicated that silent cerebrovascular disease primarily influences the domains of executive function and information processing speed, but that memory abilities remain relatively well preserved. However, memory‐related cognitive functions are known to decline in the early stages of some neurodegenerative disorders such as Alzheimer's disease (AD). Lene et al. investigated the relations between WMH burden and distribution of WMH, amyloid pathology, and vascular risk. That study showed that posterior WMH burden was associated with higher levels of amyloid beta 1–42 in both normal subjects and in patients with mild cognitive impairment, whereas frontal WMHV was associated with cardiovascular risk.^33^ It has been reported that the cerebroarterial pulsatility index and resistivity index of the internal carotid artery are negatively associated with cognitive performance and positively correlated with WMHV.^34^ Although vascular pathologies, such as blood–brain barrier breakdown and arterial stiffness, have been regarded as important factors contributing to the cognitive deficits observed in neurodegenerative diseases such as AD,^35^, ^36^ little is known about how cognitive decline might differ among patients suffering from cerebrovascular or neurodegenerative diseases. The differences may be related to the pathophysiological basis of the diseases. For neurodegenerative diseases such as AD, the pathological manifestation is atrophy of the temporal lobe hippocampus, which is highly likely to affect memory‐related functions. Therefore, it is not unexpected that AD patients may show memory‐related cognitive impairment even in the early stage of the disease.

In our study, we showed that the integrity of the IFOF, ATR, and SLF partly mediated the effect of WMHs on executive function and information processing speed. The IFOF is one of the longest nerve fibers in the brain, connecting the fronto‐marginal gyrus and lateral orbito‐frontal gyrus with the inferior occipital gyrus, the inferior part of the middle occipital gyrus, and the lingual gyrus.^29^ Its function is not clear, but it has been suggested to play an important role in executive function.^37^ The findings of a recent study showed that the IFOF and the fronto‐parietal white matter of the genu of the corpus collosum mediate the association between age and executive function in healthy aging and that the WMH burden within these white matter tracts influences this association.^38^ This implies that the related fibers may play an important role as carriers underlying brain‐behavior associations.

Studies have found that ATR damage occurs in early AD patients, and this white matter damage is not accompanied by changes in gray matter.^39^ As an important part of the forelimb of the internal capsule, the ATR includes two main parts, including the fibers from the dorsomedial nucleus of the thalamus to the frontal cortex and the fibers between the anterior thalamic nucleus and the anterior cingulate cortex.^40^ The disorder in information exchange between the dorsomedial thalamic nucleus and the frontal cortex may affect information processing speed.^41^ As a large association fiber tract, the SLF connects cortical areas of various lobes with a wide range of functions including information processing, executive function, language function, and emotional regulation.^42^ The integrity of the SLF was consistently shown to be impaired in mental disorders.^43^ Spitz and colleagues showed that information processing speed is related to an extensive network of tracts throughout the brain, including the left and right SLF. The underlying mechanism involved in the information processing speed of initiation and response inhibition is related to reduced integrity in the SLF.^44^

A previous study by Vergoossen et al. showed that WMHs and information processing speed are positively correlated and that local efficiency plays a mediating role in this correlation. In the population they studied, the periventricular WMHV was larger than the deep WMHV.^45^ This might imply that periventricular WMHs affect information processing speed more easily, which is consistent with the findings of our current study. However, Leonie et al. reported that WMHV was negatively correlated with executive function, memory, and motor speed performance, which was not completely consistent with our research results. Their study further investigated the WMH location‐specific impact on cognition, and their results showed that frontal WMH in the proximity of the frontal ventricles mainly affect executive function, that parieto‐temporal WMH in the proximity of the posterior horns deteriorates memory, and that WMH in the upper deep white matter mainly affects motor speed performance.^46^ In the current study, the prevalence of periventricular WMHs was greater than that of deep WMHs according to the WMH overlap map. Because the association tracts such as the IFOF and SLF cross the anterior and posterior horns, which contain regions with a high WMH prevalence, periventricular WMHs are more likely to damage the integrity of association tracts. Our current study also found that projection tracts such as the ATR partially mediated the correlation between WMHs and information processing speed. The ATR is formed by fibers interconnecting the anterior and medial thalamic nuclei and the cerebral cortex of the frontal lobe via the anterior limb of the internal capsule. This tract traverses regions with a certain distribution of white matter lesions that are mainly located in the subcortical structures of the frontal and parietal lobe. Greater WMHV has been associated with executive function via white matter disruption in commissural fibers,^47^ which was not in line with the findings of our study. The commissural tracts such as the CFM and CFJ traverse the regions with relatively lower prevalence of WMH in our current study, and the preservation of the integrity of the commissural traces might be one of the reasons why we failed to detect a significant correlation between the FA values of the commissural tracts and cognitive function in the current study population. The differences in these results may imply that the location of WMHs matters. Damage to brain structures leads to abnormal brain function, and the pathophysiological characteristics of WMH may contribute to a decline in the integrity of fiber tracts in specific brain regions and might lead to specific dysfunctions, including cognitive impairment.

Several limitations of our study should be addressed. First, due to the limitations of time and conditions, the sample size of this cross‐sectional study is relatively small, thus whether the results from our study are representative is yet to be confirmed by further research. Second, we calculated the volume of whole brain gray matter instead of that of specific relevant cognitive brain areas such as the hippocampus, prefrontal cortex, or thalamus. This was because we wondered whether the cognitive function in WMH patients without overt cognitive decline was related to the whole cortical atrophy caused by Wallerian degeneration after white matter lesions rather than in specific local brain areas. Third, the average age (56.14 years) of the population included in this study is relatively young, and thus we should be cautious when the conclusions of this study are applied to the elderly. Finally, we analyzed the integrity of white matter tracts as measured by FA in order to identify the role that white matter microstructure changes in WMH have on cognitive performance. Further studies are necessary to examine which kinds of white matter injury (demyelinating changes or axonal damage or both) have critical effects on cognitive function.

## 5 CONCLUSION

In summary, we found that executive function and information processing speed may be associated with WMH burden in patients with silent cerebrovascular diseases, and we propose that this association may depend on the integrity of the white matter tracts. These findings suggest a comprehensive picture of WMH's effects on cognitive function, and they might be helpful in forming the theoretical basis for rehabilitation strategies of cognitive function in patients with silent cerebrovascular diseases.

## CONFLICT OF INTEREST

There are no conflicts of interest that need to be disclosed.

## Data Availability

The data that support the findings of this study are available on request from the corresponding author. The data are not publicly available due to privacy or ethical restrictions.

## APPENDIX 1

**TABLE A1:** Successful identification rate for each of 20 fiber tracts among 236 participants.

Index	Name of tract	Number of participants showing successful tract identification	Ratio
1	Left ATR	218	0.92
2	Right ATR	216	0.91
3	Left CST	216	0.91
4	Right CST	216	0.91
5	Left CC	174	0.74
6	Right CC	168	0.71
7	Left CH	172	0.73
8	Right CH	176	0.75
9	CFM	213	0.90
10	CFJ	213	0.90
11	Left IFOF	208	0.88
12	Right FIOF	206	0.87
13	Left ILF	211	0.89
14	Right ILF	212	0.90
15	Left SLF	218	0.92
16	Right SLF	218	0.92
17	Left UF	213	0.90
18	Right UF	212	0.90
19	Left AF	205	0.87
20	Right AF	203	0.86

Abbreviations: AF, Arcuate Fascicle; ATR, Anterior Thalamic Radiation; CC, Cingulum Cingulate; CFJ, Callosum Forceps Minor; CFM, Callosum Forceps Major; CH, Cingulum Hippocampus; CST, Corticospinal tract; IFOF, Inferior Fronto‐ occipito Fasciculus; ILF, Inferior Longitudinal Fascicle; SLF, Superior Longitudinal Fascicle; UF, Uncinate Fascicle.

## APPENDIX 2

**TABLE A2:** The results of mediating model test of WMHV on executive function

Outcome	*R*	*R* ^2^	*F*	Coefficient	*T*	*p*
FA of right IFOF						
Constant	0.310	0.096	2.215	0.274	1.748	0.082
Log_10_^(WMHV)^				−0.055	−3.218	0.002
Sex				0.017	0.628	0.531
Age				−0.001	−0.551	0.582
Educational level				0.005	1.401	0.163
Hypertension				−0.022	−1.121	0.264
Diabetes mellitus				0.009	0.407	0.685
Dyslipidemia				−0.008	−0.361	0.719
BMI				0.002	0.660	0.510
Smoking				0.026	1.343	0.181
Drinking				−0.028	−1.082	0.281
Executive function						
Constant	0.506	0.256	7.148	−0.545	−0.823	0.411
Log_10_^(WMHV)^				−0.199	−3.015	0.003
Sex				0.318	2.927	0.004
Age				−0.015	−2.651	0.009
Educational level				0.041	4.291	0.000
Hypertension				−0.013	−0.155	0.877
Diabetes mellitus				−0.018	−0.144	0.886
Dyslipidemia				0.138	1.756	0.081
BMI				−0.005	−0.369	0.713
Smoking				−0.039	−0.409	0.683
Drinking				0.030	0.329	0.742
Executive function						
Constant	0.536	0.287	7.591	−0.767	−1.184	0.238
FA of right IFOF				0.808	3.827	0.003
Log_10_^(WMHV)^				−0.154	−2.381	0.024
Sex				0.304	2.873	0.005
Age				−0.015	−2.565	0.011
Educational level				0.037	3.995	0.000
Hypertension				0.004	0.053	0.958
Diabetes mellitus				−0.025	−0.207	0.836
Dyslipidemia				0.144	1.853	0.065
BMI				−0.006	−0.488	0.626
Smoking				−0.060	−0.634	0.527

Abbreviations: BMI, body mass index; FA, fractional anisotropy; IFOF, interior frontal occipital fasciculus; WMHV, white matter hyperintensity volume.

## APPENDIX 3

**TABLE A3:** The results of mediating model test of WMHV on information processing speed.

Outcome	*R*	*R* ^2^	*F*	Coefficient	*T*	*p*
FA of right ATR						
Constant	0.416	0.173	4.047	−0.208	−0.757	0.450
Log_10_^(WMHV)^				−0.151	−4.701	0.000
Sex				0.097	1.853	0.065
Age				0.001	0.540	0.590
Educational level				0.010	2.462	0.015
Hypertension				−0.032	−0.839	0.403
Diabetes mellitus				0.051	1.190	0.236
Dyslipidemia				0.039	1.043	0.298
BMI				−0.004	−0.677	0.499
Smoking				0.083	1.561	0.120
Drinking				−0.008	−0.219	0.827
FA of left SLF						
Constant	0.287	0.082	1.708	0.297	1.349	0.179
Log_10_^(WMHV)^				−0.062	−2.308	0.022
Sex				0.022	0.557	0.579
Age				−0.001	−0.850	0.397
Educational level				0.005	1.310	0.192
Hypertension				−0.027	−1.264	0.208
Diabetes mellitus				−0.038	−2.094	0.038
Dyslipidemia				0.012	0.323	0.747
BMI				−0.002	−0.508	0.612
Smoking				0.031	1.053	0.294
Drinking				−0.045	−1.015	0.312
Information processing speed						
Constant	0.436	0.190	4.562	−2.417	−3.223	0.002
Log_10_^(WMHV)^				−0.407	−4.346	0.000
Sex				0.350	2.506	0.013
Age				0.003	0.451	0.653
Educational level				0.043	3.310	0.001
Hypertension				0.025	0.253	0.801
Diabetes mellitus				0.169	1.412	0.160
Dyslipidemia				0.154	1.488	0.138
BMI				−0.011	−0.718	0.474
Smoking				0.092	0.769	0.443
Drinking				0.065	0.511	0.610
Information processing speed						
Constant	0.510	0.260	5.619	−2.462	−3.125	0.002
FA of right ATR				0.656	3.263	0.001
FA of left SLF				0.613	2.538	0.012
Log_10_^(WMHV)^				−0.270	−2.813	0.005
Sex				0.274	2.071	0.040
Age				0.003	0.450	0.653
Educational level				0.033	2.743	0.007
Hypertension				0.062	0.617	0.538
Diabetes mellitus				0.159	1.338	0.183
Dyslipidemia				0.121	1.199	0.232
BMI				−0.008	−0.524	0.601
Smoking				0.018	0.144	0.885
Drinking				0.098	0.773	0.440

Abbreviations: ATR, anterior thalamic radiation; BMI, body mass index; FA, fractional anisotropy; SLF, superior longitudinal fasciculus; WMHV, white matter hyperintensity volume.

## APPENDIX 4

**TABLE A4:** Linear regression analysis for executive function and information processing speed for averaged the middle 60% of each tract to create a single estimate of diffusion properties for each subject and tract.

Variables	Unstandardized coefficient	Standardized coefficient	*T*	Boot 95% CI		*p* value	VIF
Executive function							
Constant	−1.838	–	5.635	−2.484	−1.193	0.000	–
Educational level	0.050	0.334	4.232	0.027	0.074	0.000	1.111
FA of right IFOF	0.670	0.190	2.427	0.124	1.217	0.017	1.095
Log_10_^(WMHV)^	0.203	−0.183	−2.373	−0.373	−0.034	0.019	1.066
Information processing speed							
Constant	−2.207	–	−6.522	−2.877	−1.538	0.000	–
Log_10_^(WMHV)^	−0.464	−0.366	−4.517	−0.668	−0.261	0.000	1.182
FA of right ATR	0.452	0.182	2.238	0.052	0.851	0.027	1.187
FA of left SLF	0.674	0.165	2.153	0.055	1.293	0.033	1.063

Abbreviations: ATR, anterior thalamic radiation; CI, confidence interval; FA, fractional anisotropy; IFOF, interior frontal occipital fasciculus; SLF, superior longitudinal fasciculus; VIF, Variance inflation factor; WMHV, white matter hyperintensity volume.

**TABLE A5:** Linear regression analysis for executive function and information processing speed for averaged the middle 100% of each tract to create a single estimate of diffusion properties for each subject and tract.

Variables	Unstandardized coefficient	Standardized coefficient	*T*	Boot 95% CI		*p* value	VIF
Executive function							
Constant	−1.836	–	5.645	−2.480	−1.193	0.000	–
Educational level	0.049	0.329	4.138	0.026	0.072	0.000	1.104
FA of right IFOF	0.672	0.193	2.436	0.126	1.218	0.016	1.097
Log_10_^(WMHV)^	0.205	−0.188	−2.413	−0.372	−0.037	0.017	1.062
Information processing speed							
Constant	−2.180	‐	−6.567	−2.837	−1.523	0.000	‐
Log_10_^(WMHV)^	−0.444	−0.357	−4.411	−0.643	−0.245	0.000	1.178
FA of right ATR	0.518	0.207	2.548	0.116	0.920	0.027	1.191
FA of left SLF	0.711	0.177	2.315	0.103	1.293	1.319	1.058

Abbreviations: ATR, anterior thalamic radiation; CI, confidence interval; FA, fractional anisotropy; IFOF, interior frontal occipital fasciculus; SLF, superior longitudinal fasciculus; VIF, Variance inflation factor; WMHV, white matter hyperintensity volume.

## APPENDIX 5

**TABLE A6:** Total effect, direct effect and mediating effect of log transformed WMH volume on executive function for averaged the middle 60% of each tract to create a single estimate of diffusion properties for each subject and tract.

	Effect size	Boot SE	Boot LLCI	Boot ULCI	Proportion of relative effect
Total effect	−0.200	0.069	−0.336	−0.064	
Direct effect	−0.145	0.068	−0.280	−0.036	72.50%
Mediating effect of FA of right IFOF	−0.055	0.023	−0.115	−0.021	27.50%

Abbreviations: FA, fractional anisotropy; IFOF, interior frontal occipital fasciculus; LLCI, Lower limit of confidence interval; SE, standard error; ULCI, Upper limit of confidence interval.

**TABLE A7:** Total effect, direct effect and mediating effect of log transformed WMH volume on executive function for averaged the middle 100% of each tract to create a single estimate of diffusion properties for each subject and tract.

	Effect size	Boot SE	Boot LLCI	Boot ULCI	Proportion of relative effect
Total effect	−0.199	0.067	−0.336	−0.067	
Direct effect	−0.154	0.068	−0.288	−0.021	77.38%
Mediating effect of FA of right IFOF	−0.045	0.019	−0.094	−0.016	22.62%

Abbreviations: FA, fractional anisotropy; IFOF, interior frontal occipital fasciculus; LLCI, Lower limit of confidence interval; SE, standard error; ULCI, Upper limit of confidence interval.

## APPENDIX 6

**TABLE A8:** Total effect, direct effect and mediating effect of log transformed WMH volume on information processing speed for averaged the middle 60% of each tract to create a single estimate of diffusion properties for each subject and tract.

	Effect size	Boot SE	Boot LLCI	Boot ULCI	Proportion of relative effect
Total effect	−0.399	0.093	−0.582	−0.215	
Direct effect	−0.271	0.095	−0.459	−0.083	68.02%
Total indirect effect	−0.128	0.048	−0.250	−0.055	31.98%
Mediating effect of FA of right ATR	−0.092	0.040	−0.196	−0.034	23.06%
Mediating effect of FA of left SLF	−0.035	0.020	−0.081	−0.001	8.92%
Effect of FA of right ATR minus FA of left SLF	−0.058	0.040	−0.006	0.151	–

Abbreviations: FA, fractional anisotropy; LLCI, Lower limit of confidence interval; SE, standard error; SLF, superior longitudinal fasciculus; TR, anterior thalamic radiation; ULCI, Upper limit of confidence interval.

**TABLE A9:** Total effect, direct effect and mediating effect of log transformed WMH volume on information processing speed for averaged the middle 100% of each tract to create a single estimate of diffusion properties for each subject and tract.

	Effect size	Boot SE	Boot LLCI	Boot ULCI	Proportion of relative effect
Total effect	−0.407	0.094	−0.591	−0.222	
Direct effect	−0.270	0.096	−0.459	−0.081	66.33%
Total indirect effect	−0.137	0.050	−0.260	−0.062	33.66%
Mediating effect of FA of right ATR	−0.099	0.042	−0.209	−0.038	24.32%
Mediating effect of FA of left SLF	−0.038	0.018	−0.083	−0.006	9.34%
Effect of FA of right ATR minus FA of left SLF	−0.061	0.041	−0.169	0.001	–

Abbreviations: FA, fractional anisotropy; LLCI, Lower limit of confidence interval; SE, standard error; SLF, superior longitudinal fasciculus; TR, anterior thalamic radiation; ULCI, Upper limit of confidence interval.
